# The COVID-19 pandemic in Singapore: what does it mean for arthroplasty?

**DOI:** 10.1080/17453674.2020.1774138

**Published:** 2020-06-08

**Authors:** Joshua Decruz, Sumanth Prabhakar, Benjamin Tze Kiong Ding, Remesh Kunnasegaran

**Affiliations:** Department of Orthopaedic Surgery, Tan Tock Seng Hospital, Singapore

## Abstract

Background and purpose — The ongoing Coronavirus Disease-19 (COVID-19) pandemic has taken a toll on healthcare systems around the world. This has led to guidelines advising against elective procedures, which includes elective arthroplasty. Despite arthroplasty being an elective procedure, some arthroplasties are arguably essential, as pain or functional impairment maybe devastating for patients, especially during this difficult period. We describe our experience as the Division of Arthroplasty in the hospital at the epicenter of the COVID-19 pandemic in Singapore.

Patients and methods — The number of COVID-19 cases reported both nationwide and at our institution from February 2020 to date were reviewed. We then collated the number of arthroplasties that we were able to cope with on a weekly basis and charted it against the number of new COVID-19 cases admitted to our institution and the prevalence of COVID-19 within the Singapore population.

Results — During the COVID-19 pandemic period, a significant decrease in the volume of arthroplasties was seen. 47 arthroplasties were performed during the pandemic period from February to April, with a weekly average of 5 cases. This was a 74% reduction compared with our institutional baseline. The least number of surgeries were performed during early periods of the pandemic. This eventually rose to a maximum of 47% of our baseline numbers. Throughout this period, no cases of COVID-19 infection were reported amongst the orthopedic inpatients at our institution.

Interpretation — During the early periods of the pandemic, careful planning was required to evaluate the pandemic situation and gauge our resources and manpower. Our study illustrates the number of arthroplasties that can potentially be done relative to the disease curve. This could serve as a guide to reinstating arthroplasty as the pandemic dies down. However, it is prudent to note that these situations are widely dynamic and frequent re-evaluation is required to secure patient and healthcare personnel safety, while ensuring appropriate care is delivered.

Tan Tock Seng Hospital (TTSH) in Singapore is a unique institution, functioning almost as a single unit with the National Centre of Infectious Disease (NCID), which has been at the epicenter of the Coronavirus Disease-19 (COVID-19) pandemic in the nation. Following the 2002–2004 Severe Acute Respiratory Syndrome (SARS) epidemic, the NCID was built in an effort to prepare Singapore for future outbreaks and to allow for continued management of patients with chronic conditions (Kurohi 2019).

The NCID handled about 70% of the screening load at the start of the COVID-19 pandemic in Singapore, a country with a population of around 5.8 million. Operational manpower required for the running of the screening centers, wards, and intensive care units in NCID had been solely derived from TTSH until April 1, 2020, after which other hospitals in Singapore began contributing manpower to aid with the crisis.

The Division of Arthroplasty, within the Department of Orthopaedic Surgery in TTSH, consists of 8 surgeons who performed an average of 84 arthroplasties, including knee replacements, hip replacements, and elective revision arthroplasties, per month prior to the COVID-19 outbreak. When evidence of community spread of the disease first surfaced on February 7, 2020, the Ministry of Health (MOH) of Singapore responded by declaring the Disease Outbreak Response System Condition (DORSCON) alert level of Orange (Ministry of Health, Singapore 2020c [last accessed April 19, 2020]). At the epicenter of the outbreak, TTSH began reassigning manpower to ensure that NCID was adequately staffed to handle the situation. At that point, one-quarter of the manpower from the Department of Orthopaedic Surgery was deployed to assist with the screening effort in NCID. Considering the available resources, the hospital management advised a reduction in the “Business As Usual (BAU)” activities to half its capacity, which included elective surgeries like arthroplasty.

Throughout this period, the Ministry of Health remained in close contact with all public health institutions to provide directives regarding the deferment of elective surgeries, with an eye on the rising incidence of COVID-19 in Singapore, and in anticipation of the potential increase in the number of patients requiring critical care (Ministry of Health, Singapore 2020a, b [last accessed April 19, 2020]).

We describe our experience in the Division of Arthroplasty at the epicenter of the COVID-19 pandemic in Singapore.

## ^Patients and methods^

This study is a retrospective review of all elective arthroplasties that were performed since the start of the COVID-19 pandemic. The number of COVID-19 cases reported both nationwide and at our institution from February 2020 to date were also reviewed. We collated the number of arthroplasties that were performed on a weekly basis and charted it against the number of new COVID-19 cases admitted to our institution and the prevalence of COVID-19 within the Singapore population.

Surgeries were performed in concordance with hospital policy: only patients with severe arthropathy with significant impairment of function were permitted to proceed with their planned surgery during this period. Surgeries were performed without jeopardizing the stock of essential items while maintaining the safety of healthcare personnel and patients. All other planned arthroplasties were cancelled.

The months of October/November 2019 were selected as a baseline reference for the volume of arthroplasties performed weekly.

### ^Funding and potential conflicts of interest^

We did not receive funding of any kind. We declare that there is no conflict, disclosure of relationship, or interests that could have a direct or potential influence or impart bias on the work.

## ^Results^

**Figure 1.**
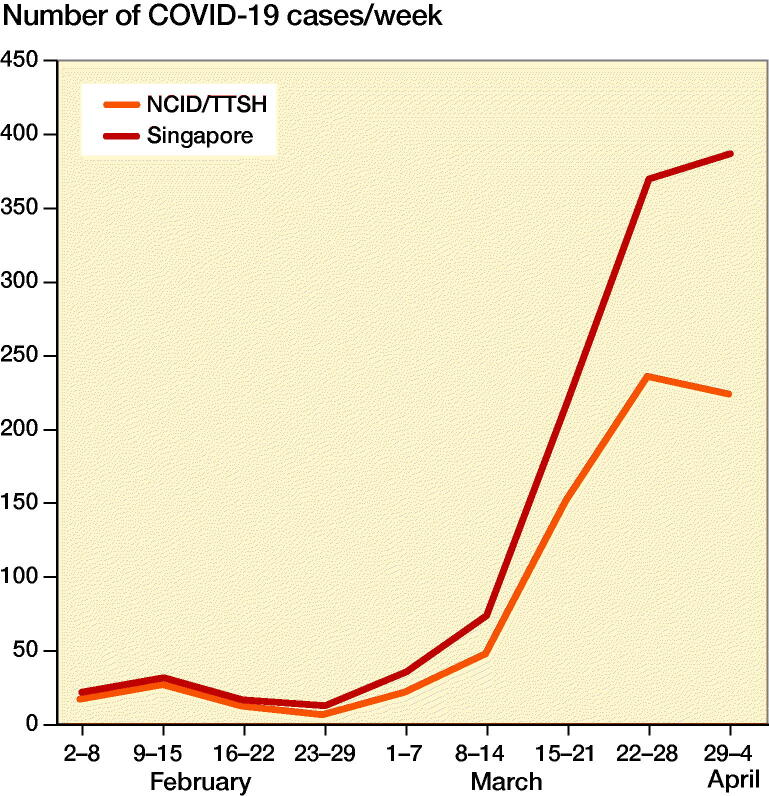
Weekly COVID-19 caseload at NCID/TTSH in comparison with cases nationwide.

**Figure 2.**
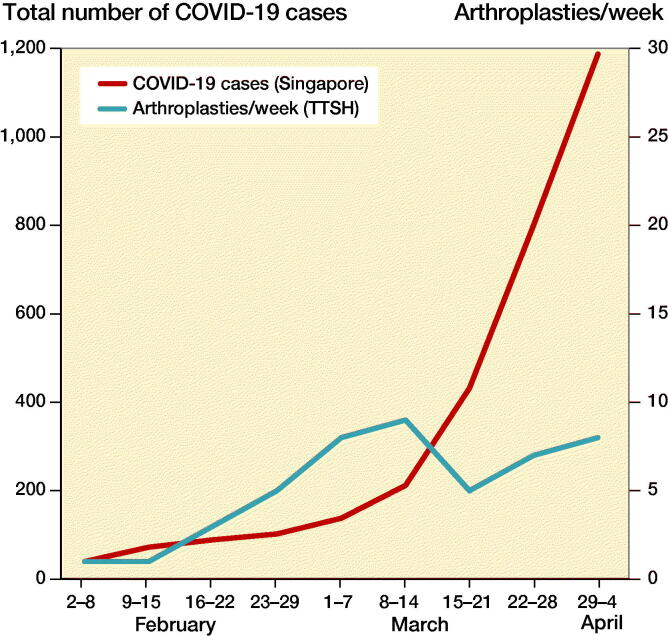
Weekly comparison of total COVID-19 cases in Singapore (cumulative numbers), and the number of arthroplasties performed at TTSH.

The majority of COVID-19 cases in Singapore were admitted to our institution (Figure 1). Of the 1,189 cases reported by April 4, 2020, 757 (64%) were admitted to both NCID and TTSH. The number of COVID-19 increased substantially after March 14, from 212 on March 14, to 1,189 on April 4 (Figure 2).

167 arthroplasties were performed during the baseline reference months of October–November 2019, which translates to an average weekly volume of 19 surgeries.

**Figure 3.**
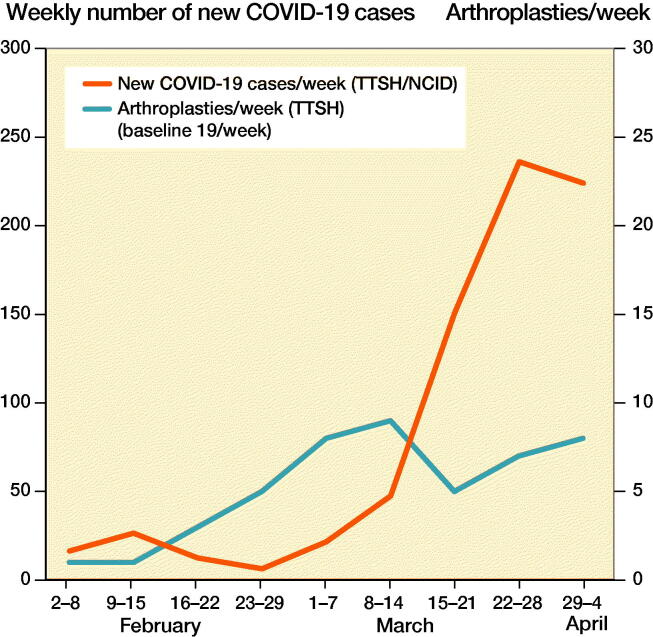
Weekly comparison of number of new COVID-19 cases reported at TTSH/NCID and number of arthroplasties performed at TTSH.

During the COVID-19 pandemic period, the volume of arthroplasties decreased (Figures 2 and 3). 47 arthroplasties were performed during the pandemic period from February to April, with a weekly average of 5 cases. This was a 74% reduction compared with our institutional baseline. The patients’ mean age was 68 years, with a mean BMI of 27. Most of them were ASA Class II (Table 1).

**Table 1.** Demographic data on 47 arthroplasties

Demographic	mean (SD)	n
Female sex		35
Age	68 (8.1)	
50–59		11
60–69		13
70–79		19
80–89		4
BMI	27 (5.5)	
15–19		2
20–24		15
25–29		19
30–34		6
35–39		3
40–44		1
45–49		1
ASA Class:		
1		1
2		32
3		14
Operation performed		
TKR		32
THR		5
UKA		10
Indication		
Osteoarthritis		39
Rheumatoid arthritis		2
Avascular necrosis		4
Revision surgery		2
Length of stay (days)	4.7 (2.3)	
		29
5–10		16
		2

A significant reduction in arthroplasties was initially seen during the early periods of the pandemic with the least number of cases being performed (Figures 2 and 3). Only 2 arthroplasties were performed between February 2 and February 15, 2020, which was a mere 5% of our usual volume. There was an increase in the number of elective arthroplasties between February 15 and April 4, 2020 with a peak volume of 9 cases weekly (47% of baseline) during the March 8 to March 14.

During the pandemic period, a review of medical records and readmissions showed that none of the orthopedic inpatients in our institution were diagnosed with COVID-19 infection. The diagnosis of COVID-19 infection was based on the results of the RT-PCR based nasopharyngeal swab.

## ^Discussion^

The ongoing Coronavirus Disease-19 (COVID-19) pandemic has taken a toll on healthcare systems around the world. Shortage of resources, such as personal protective equipment (PPE), masks, ventilators, ICU beds, and even adequately trained healthcare workers has made this pandemic a global crisis (Vannabouathong et al. 2020). Despite being a purpose-built center, the National Centre of Infectious Disease (NCID) in Singapore has now reached its full capacity. We now cope with a progressively lower proportion of the total number of patients in Singapore from a peak of 78% in mid-February to 64% as of April 4 (Figure 1). The other hospitals and community isolation facilities in Singapore have ramped up their efforts in handling the pandemic.

In our institution, the initial reduction in the number of arthroplasties performed between February 2 and February 15, 2020 coincided with the referral of all potential COVID-19 cases from primary healthcare to NCID and a resultant diversion of manpower from TTSH to NCID. This planned reduction of caseload also allowed the institution to plan effectively for the evolving pandemic. We worked with the hospital administrators and anesthetists to evaluate the pandemic and gauge our resources and manpower.

The number of surgeries performed increased marginally after February 15, 2020 with the hospital administration allowing for arthroplasties in patients with severe arthropathy to proceed because of the initial low and controlled numbers of patients with COVID-19. A summary of the management goals, manpower reorganization, and elective caseload limits of our Arthroplasty Division is shown against the World Health Organization (WHO) pandemic phases in Table 2 (World Health Organization 2010).

**Table 2.** Management of TTSH Arthroplasty Division against the World Health Organization (WHO) pandemic phases

WHO pandemic phase	Definition	Arthroplasty unit management goals	Arthroplasty caseload limit	Manpower contribution for pandemic	Elective cases selection criteria	


**Phase 3**	Human infection (transmission in close contacts)	Assessment of stockpile and resources	100%	0%	All cases	
**Phase 4**	Small cluster (< 25 cases lasting < 2 weeks)	Consider postponing future cases that are high risk (ASA 3 and above) or less severe	100%	0%	All cases	
**Phase 5**	Large cluster (25–50 cases over 2–4 weeks) usage	Minimize use of intensive care beds/blood products Limit stockpile and manpower Severe arthropathy with limited mobility	50%	25%	ASA 1 or 2 patients Unlikely to require intensive care/blood products	



**Phase 6**	Widespread in general population	Preserve intensive care beds/blood products Stockpile for pandemic usage solely	0%	25–75%	Cancel all cases	
**Post-peakperiod**	Levels of infections have dropped below peak levels in most countries	Minimize use of intensive care beds/blood products Limit stockpile and manpower usage	25%	25%	Same as Phase 5	

With efforts concentrated on the control and eradication of this infection, the management of patients with unrelated chronic diseases, including severe arthropathies, has taken a step back and rightfully so. Guidelines have been developed by institutions to aid surgeons with their practice and with prioritization of elective surgeries. The main aims of these guidelines are: (1) to minimize use of essential items (PPE, cleaning supplies, ventilators, bed, blood products, drugs, and personnel); (2) protect healthcare personnel and patients from additional risks during this period; and (3) ensure appropriate care is delivered (Ding et al. 2020, Guy et al. 2020, Liang et al. 2020b, Soh et al. 2020, Vannabouathong et. al 2020).

The American College of Surgeons (ACS) Guidance for Elective Surgery recommended that all surgeries for chronic hip and knee pain be rescheduled in the early stages of the pandemic. In the later stages, even surgeries for acute hip and knee pain were to be rescheduled (American College of Surgeons [last accessed April 19, 2020]). On the other hand, the National Health Service (NHS) of the United Kingdom suggested that elective surgery can proceed during low COVID-19 prevalence, especially for ASA class 1 patients (National Health Service, United Kingdom [last accessed April 19, 2020]). Similarly, the algorithm from Piedmont Orthopedics in Georgia, USA suggested that elective surgeries can proceed after careful consideration of all factors (Schmidt 2020). The American Academy of Orthopaedic Surgeons (AAOS) also suggests the application of guidelines depending on the severity of the pandemic in each country or area and their available resources (Guy et al. 2020). The WHO recommends that routine and elective services be deferred immediately or displaced to other settings or non-affected areas. However, this is to be done while minimizing adverse effects of such interruptions, such as decreased quality of life, increased burden on caregivers, and poor self-management as a result of an exacerbation of existing conditions (Regional Office for Europe, World Health Organization [last accessed May 11, 2020]).

However, proceeding with elective surgeries that use essential items has been said to be shortsighted and negatively looked upon in the media, especially when the availability of these items may be unequal between institutions and regions (Guy et al. 2020, O’Donnell 2020). Only 5 of the 50 states in the United States of America provided guidance on elective orthopedic procedures, and 4 out of these 5 states advised against all arthroplasty procedures during this period (Sarac et al. 2020). Subsequently, Liang et al. (2020a) recommend not exceeding 25% of the usual caseload during the post-peak period of the pandemic, as there will still be a need for evaluation of response, recovery, and preparation for a possible second wave.

Arthroplasty has been one of the most commonly performed elective surgical procedures and has been increasing in incidence in a linear fashion in recent times (Sloan et al. 2018). The average number of arthroplasties in the Organisation for Economic Co-operation and Development (OECD) countries in 2015 was 292 per 100,000 population (OECD 2017). This is because osteoarthritis is recognized as 1 of the 10 most disabling diseases in developed countries. Despite being an elective procedure, arguments can be made for the need for some arthroplasties, as pain or functional impairment could prove devastating for patients, especially during this difficult period (Ding et al. 2020). The 47 arthroplasties performed in our institution were selected on that basis in accordance with our institutional guidelines.

Our study illustrates the number of arthroplasties that can be done relative to the disease curve. As an institution, it may be possible for us to reinstate arthroplasties as the pandemic dies down based on these numbers, provided that the resources and manpower remain available. In case of subsequent waves of this pandemic, we may also use these numbers as a guide to cut down on elective arthroplasties in a timely manner.
